# Severe congenital neutropenia: case report

**DOI:** 10.1186/1939-4551-8-S1-A277

**Published:** 2015-04-08

**Authors:** Luiz Carlos Bandoli Gomes

**Affiliations:** 1 HU-Ufjf, Brazil

## Introduction

Neutropenia is defined in the literature as absolute neutrophil counts in peripheral blood of less than 1500 cells/mm3 in more than one year old and less than 2000cells/mm3 in children in the first year old of life. Neutropenia is classified as mild, moderate or severe, and may be congenital or acquired, persistent or not. Kostmann syndrome is a severe neutropenia, the incidence varies1-2 cases/ 100.000 – 1.000.000 and attends with severe recurrent infections early.

## Case description

JBL, white, male was admitted three times with recurrent pneumonia, otitis, anemia, neutropenia, eosinophilia and monocytose in peripheral blood and cord lock maturation phase pro-myelocytic to the bone marrow. Used G-CSF. At 11 months od age showed severe pneumonia without clinical response and death.

## Discussion

recurrent infection in this child began early, as happen in monocytosis, lymphocytosis and eosinophilias associated with maturation arrest of marrow in the series promyelocytic suggest the diagnosis of syndrome Kostmann. Beside the proper treatment of infections is indicated using G-CSF.

## Conclusions

Kostmann syndrome should be considerate in the differential diagnosis of severe persistent neutropenia in children, among the prophylaxis and treatment of infections associated with the use of G-CSF members of appropriate monitoring of the patients.

## Consent

Written informed consent was obtained from the patient for publication of this abstract and any accompanying images. A copy of the written consent is available for review by the Editor of this journal.
